# Co-solvation effect on the binding mode of the α-mangostin/β-cyclodextrin inclusion complex

**DOI:** 10.3762/bjoc.11.251

**Published:** 2015-11-25

**Authors:** Chompoonut Rungnim, Sarunya Phunpee, Manaschai Kunaseth, Supawadee Namuangruk, Kanin Rungsardthong, Thanyada Rungrotmongkol, Uracha Ruktanonchai

**Affiliations:** 1 National Nanotechnology Center (NANOTEC), National Science and Technology Development Agency (NSTDA), Pathumthani 12120, Thailand; 2 Faculty of Pharmacy Thammasat University, Rangsit Center, Pathumthani 12120 Thailand; 3 Structural and Computational Biology Unit, Department of Biochemistry, Faculty of Science, Chulalongkorn University, Bangkok 10330, Thailand; 4 Ph.D. Program in Bioinformatics and Computational Biology, Faculty of Science, Chulalongkorn University, Bangkok 10330, Thailand

**Keywords:** α-mangostin, β-cyclodextrin, binary complex, inclusion complex

## Abstract

Cyclodextrins (CDs) have been extensively utilized as host molecules to enhance the solubility, stability and bioavailability of hydrophobic drug molecules through the formation of inclusion complexes. It was previously reported that the use of co-solvents in such studies may result in ternary (host:guest:co-solvent) complex formation. The objective of this work was to investigate the effect of ethanol as a co-solvent on the inclusion complex formation between α-mangostin (α-MGS) and β-CD, using both experimental and theoretical studies. Experimental phase-solubility studies were carried out in order to assess complex formation, with the mechanism of association being probed using a mathematical model. It was found that α-MGS was poorly soluble at low ethanol concentrations (0–10% v/v), but higher concentrations (10–40% v/v) resulted in better α-MGS solubility at all β-CD concentrations studied (0–10 mM). From the equilibrium constant calculation, the inclusion complex is still a binary complex (1:1), even in the presence of ethanol. The results from our theoretical study confirm that the binding mode is binary complex and the presence of ethanol as co-solvent enhances the solubility of α-MGS with some effects on the binding affinity with β-CD, depending on the concentration employed.

## Introduction

Solubilization of otherwise poorly soluble drugs under physiological conditions to improve their bioavailability is challenging, and a requirement for the design and development of effective formulations. There are several ways to favorably enhance the solubility of poorly soluble drugs which include micronization, chemical modification, pH adjustment, complexation [1], co-solvent addition [2–8] and surfactant addition [9]. Complexation is one of the most utilized methods for enhancing the solubility of poorly soluble drugs. Cyclodextrins (CDs) are well-known macrocyclic oligosaccharides that are produced by enzymatic degradation of starch. CDs consist of 6, 7 and 8 α-D-glucopyranose units and are depicted as α-CD, β-CD and γ-CD, respectively. CDs are able to bind non-polar molecules, including poorly soluble drugs, in their hydrophobic cavities to form binary inclusion complexes [10–12]. Inclusion of the drug can result in its enhanced solubility, dissolution rate, bioavailability, and stability (in comparison to the free drug), with controlled release also being possible [13–16]. In addition, co-solvent addition is a well-established method for increasing the equilibrium solubility of non-polar drugs. Recent studies combining co-solvent addition with complexation [2–3,6] have demonstrated that the thermodynamics underlying the interactions between host–guest molecules can be significantly changed in these instances. In these cases, the co-solvent can also occupy the CD cavity in conjunction with the guest (drug) molecules to form CD/guest/co-solvent ternary complexes. In other studies, the co-solvent has been shown to compete with the drug molecules for the entry into the CD cavity, with the result of lower drug loadings (inclusion of drug molecules) in the system. Besides, the co-solvent effect was found as a factor that control anion affinity and selectivity of a neutral anion receptor, bis(cyclopeptide) [17].

Molecular dynamics (MD) simulations can give important insights into the energetics of structural interactions. The hydrated structure of β-CD in aqueous solution [18] and those showing host–guest interactions between the β-CD structure and guest molecules in its inclusion compounds have been reported [19–21]. Moreover, MD simulations of β-CD in water and ethanol mixtures have been performed to investigate the orientation of the co-solvent in the hydrophobic cavity of the β-CD [22]. Recently, Biedermann et al. [23] reviewed the hydrophobic effect of supramolecular complexes from MD simulation studies and emphasized that the non-covalent driving force of high-energy water in the cavity of cyclodextrins, cyclophanes and cucurbiturils was an essential factor for complexation with the guest molecule. MD simulations are therefore a useful technique providing details of the molecular interactions of structural components in different environments (e.g., water or water/co-solvent mixtures) which are often encountered in formulations.

In our previous work [24], the preliminary results of phase solidities of the inclusion complex in ethanol and methanol were reported but the co-solvation effects was not clearly stated. Hence, to fulfill the understanding of such effects, further details of the solvation effects are presented in this work. We experimentally and theoretically study the influence of ethanol as a co-solvent on the complex formation between α-mangostin (α-MGS) and β-CD. Phase solubility studies were carried out in order to assess the formation of those complexes at various β-CD concentrations, with ethanol as a co-solvent. A simple mathematical model was then applied to explain the solubility of α-MGS influenced by the presence of β-CD and ethanol. MD simulations were performed to quantify the strength of inclusion complex formation in terms of binding energy, hydrogen bonding interactions, and displacement analysis.

## Materials and methods

### Experimental study

#### Chemicals and reagents

α-Mangostin (purity >90%), isolated from mangosteen pericarp, was obtained from Honsea Sunshine Biotech Co., Ltd. (Guangzhou, China). The α-mangostin reference standard (≥98% purity) was obtained from Sigma-Aldrich (USA). β-CD (Cavamax^®^ W7, pharmaceutical grade, purity >98%) was obtained from Wacker Chemie AG (Bangkok, Thailand). Acetic acid, ethanol, methanol, and acetonitrile were of analytical grade and supplied by Carlo Erba (Rome, Italy). Deionized (DI) water was produced using a Milli-Q Plus system (Millipore, Schwalbach, Germany).

#### Phase solubility

The phase solubility study was conducted using the Higuchi and Connors method (Higuchi and Connors, 1965). Briefly, α-mangostin (2 g, excess) was added into gas-tight vials containing both β-CD and ethanol. The concentration of β-CD (0 to 10 mM), and ethanol (0 to 40% v/v) was varied in each vial such that a series was produced. The gas-tight vials were shaken using a shaking incubator (Vision Scientific Co., Ltd., Korean) at 25 °C for 48 h to ensure equilibrium was reached. The samples were then passed through a 0.45 µm Nylon filter, and the concentration of dissolved α-mangostin was determined by high-performance liquid chromatography, HPLC, (Waters, model e2695, USA) using the following method. The photodiode array (PDA) detector was set to monitor at a λ_max_ of 320 nm. The chromatographic separation was performed at 25 °C using a C_18_ column (Waters, 250 mm × 4.6 mm, 5 µm). The eluents were composed of 1% acetic acid in DI water as a mobile phase A, and methanol as mobile phase B. The isocratic steps of A and B were set at 10% and 90%, respectively, for 15 min with a total flow rate of 1 mL/min. Sample injection volume was 10 µL. The calibration curve was made at concentrations ranging from 0.001 to 0.1 mg/mL. All samples were prepared in triplicate.

The total drug solubility in the presence of both co-solvent and CD was determined according to Equation 1:

[1]
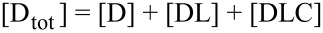


where [D_tot_] is the total solubility of the drug, [D] is the concentration of the free drug, [DL] is the concentration of the binary complex, and [DLC] is the concentration of the ternary complex.

According to Equation 2, the concentration of the free drug, [D], can be calculated based on the assumption that complexation has a negligible effect on the amount of free drug. The logarithm of the drug solubility increases linearly with the concentration of co-solvent as described in Equation 3 and co-solvent solubilizing power (σ), the slope of this linear function, depends upon the polarity of both the solute and the solvent.

[2]
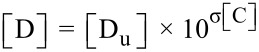


[3]
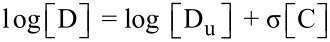


D_u_ represents the intrinsic drug solubility, σ is the co-solvent solubilizing power and [C] is the co-solvent concentration.

The concentration of the α-MGS/β-CD binary complex [DL] is directly related to the concentration of free α-MGS [D], the concentration of β-CD, and the apparent binary complexation constant, *K*_b_^app^, which can be determined according to Equation 4.

[4]
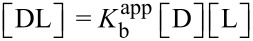


Furthermore, the apparent binary complexation constant, *K*_b_^app^, has an association with the co-solvent concentration [C], the intrinsic complexation constant, *K*_b_^int^, and co-solvent destabilizing power for the binary complex, ρ_b_, and can be determined according to Equation 5.

[5]
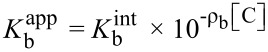


The concentration of the α-MGS/β-CD/ethanol ternary complex [DLC] is related to the concentration of free α-MGS [D], the concentration of β-CD [L], the co-solvent concentration [C] and the apparent ternary complexation constant, *K*_t_^app^, as shown in Equation 6.

[6]
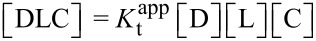


*K*_t_^app^ has a correlation with co-solvent destabilizing power for the ternary complex (ρ_t_), the intrinsic ternary complexation constant (*K*_t_^int^) and the concentration of co-solvent [C], as defined in Equation 7.

[7]
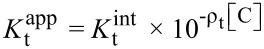


From Equation 1, the expression for total solubility of drug [D_tot_] in the presence of both co-solvent and complexation can be rearranged and expressed as in Equation 8.

[8]
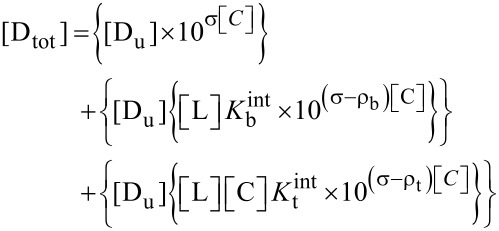


### Theoretical study

#### Structural preparation for MD simulation

**Figure 1:**
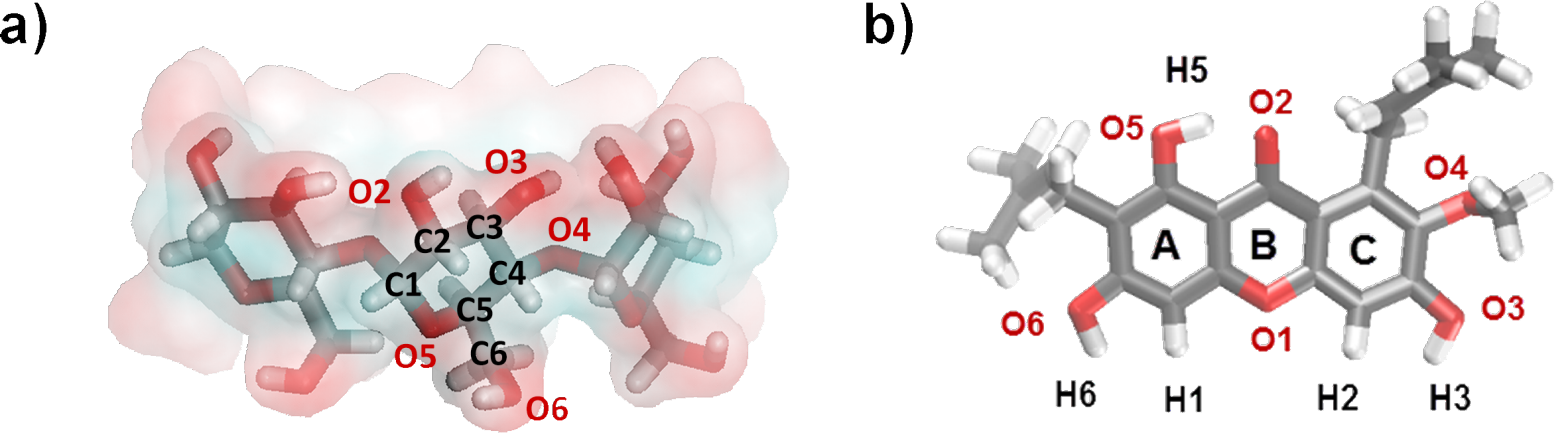
Schematic views of a) β-CD and b) α-mangostin (α-MGS) geometries.

The initial geometry of β-CD was obtained from the PDB databank (3C6G), while the α-MGS structure was extracted from the International Union of Crystallography (KP2293) database, see Figure 1. According to the ChemAxon method [25–27], the calculated p*K*_a_ of three hydroxy groups are 7.4 (O6), 7.8 (O3), and 8.2 (O5). For that reason, the α-mangostin was considered as neutral molecule in the MD simulation (pH 7). Parameters for the β-CD were applied using the Glycam-06 force field while the atomic charges and parameters for α-MGS were derived using a restrained electrostatic potential (RESP) charge-fitting procedure as described in the previous studies [28–31]. Details of atom types and partial atomic charges of α-mangostin are enclosed in Supporting Information File 1 (Table S1). The RESP was calculated at the HF/6-31G(d) level of theory using Gaussian 09 [32]. The hydrogen atoms added by the Leap module were minimized by 1000 steps of steepest descent, and followed by 2000 steps of conjugated gradients to remove bad contacts. Then, the relaxed inclusion complexes were solvated by the TIP3P water molecules with a set distance of 15 Å from the system surface. In aqueous solvation, the system consists of 3,100 water molecules within a 45.0 × 45.0 × 45.0 Å^3^ truncated periodic box. The periodic box size was kept constant for the inclusion complex at all ethanol (EtOH) concentrations (% v/v). Following this, the solvation molecules were added to the solvation box using the PACKMOL package [33]. The number of water and ethanol molecules is given in Table 1.

**Table 1:** Number of co-solvent molecules in the six simulation systems.

System	No. of water	No. of EtOH

Water	3,100	–
5% v/v EtOH	2,945	48
15% v/v EtOH	2,635	144
30% v/v EtOH	2,170	287
60% v/v EtOH	1,240	574
EtOH	–	957

#### Details of molecular dynamics simulations

In the present study, all MD simulations were performed using the SANDER module of the Amber10 software package in accordance with the recently reported MD simulations of flavonoid/β-CD inclusion complexes in water [34–35]. The particle-mesh Ewald method with a cut-off distance of 12 Å was employed. The integration time step was 2 fs and the SHAKE algorithm was applied to constrain all bonds attached to hydrogen atoms. Prior to heating, the solvent molecules were only minimized using 3,500 steps of conjugated gradients. The whole system was then heated to 300 K within the 500 steps of relaxation time using the Canonical Ensemble (NVT) algorithm at constant volume up to 1 g/mL of water density. Finally, the MD simulations were performed at 1 atm and 300 K for 20 ns.

The structural dynamics over simulation time were monitored by root mean square displacement (RMSD). The orientation and solvation of α-MGS occupying the β-CD cavity were investigated in terms of structural properties, and the radial distribution function (RDF). The hydrogen bond interactions between α-MGS and β-CD molecules were analyzed using the criteria of (i) distance between the hydrogen donor and acceptor atoms being ≤3.5 Å; and (ii) the angle of the donor-hydrogen-acceptor being ≥120° [36].

#### Binding free energy calculations

Herein, the binding free energies of α-MGS/β-CD complex were calculated as follows. The Δ*G* is defined by

[9]
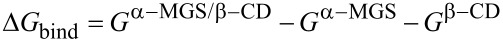


where each free energy is estimated from

[10]
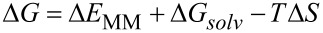


The gas phase energy, Δ*E*_MM_, is a summation of bonded and non-bonded (electrostatic and van der Waals (vdW)) energies obtained from molecular mechanics calculation. The Δ*G*_solv_ is solvation free energy. In general, there are several methods for Δ*G*_solv_ prediction. Some methods calculate the Δ*G*_solv_ using implicit solvent models such as Generalized Born (GB) [37–38], Poisson–Boltzmann (PB) [39–40] and Reference Interaction Site Model (RISM) [41]. Meanwhile, the other methods such as linear interaction energy (LIE) [42–44] and linear response approximation (LRA) [45–47] calculate the Δ*G*_solv_ based on a modified linear response to treat electrostatic interactions with an empirical term treating the dispersion interactions. In this work, the Δ*G*_solv_ was considered as polar and non-polar solvation terms. The polar solvation term is evaluated from the Poisson–Boltzmann (PB) solvation method which is successfully applied in other biological systems [29,34–35,48]. The non-polar contribution is calculated by the solvent-assessable surface area (SASA) as

[11]
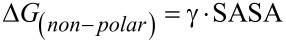


Where γ was set as 0.0072 kcal/(mol/A^2^) [49]. The *TS* term is a solute entropy contribution arising from changes in degrees of freedom (translation, rotation and vibration) of the molecule which can be estimated using the NMODE module in Amber10.

## Results and Discussion

### Experimental results

#### Phase solubility results

**Figure 2:**
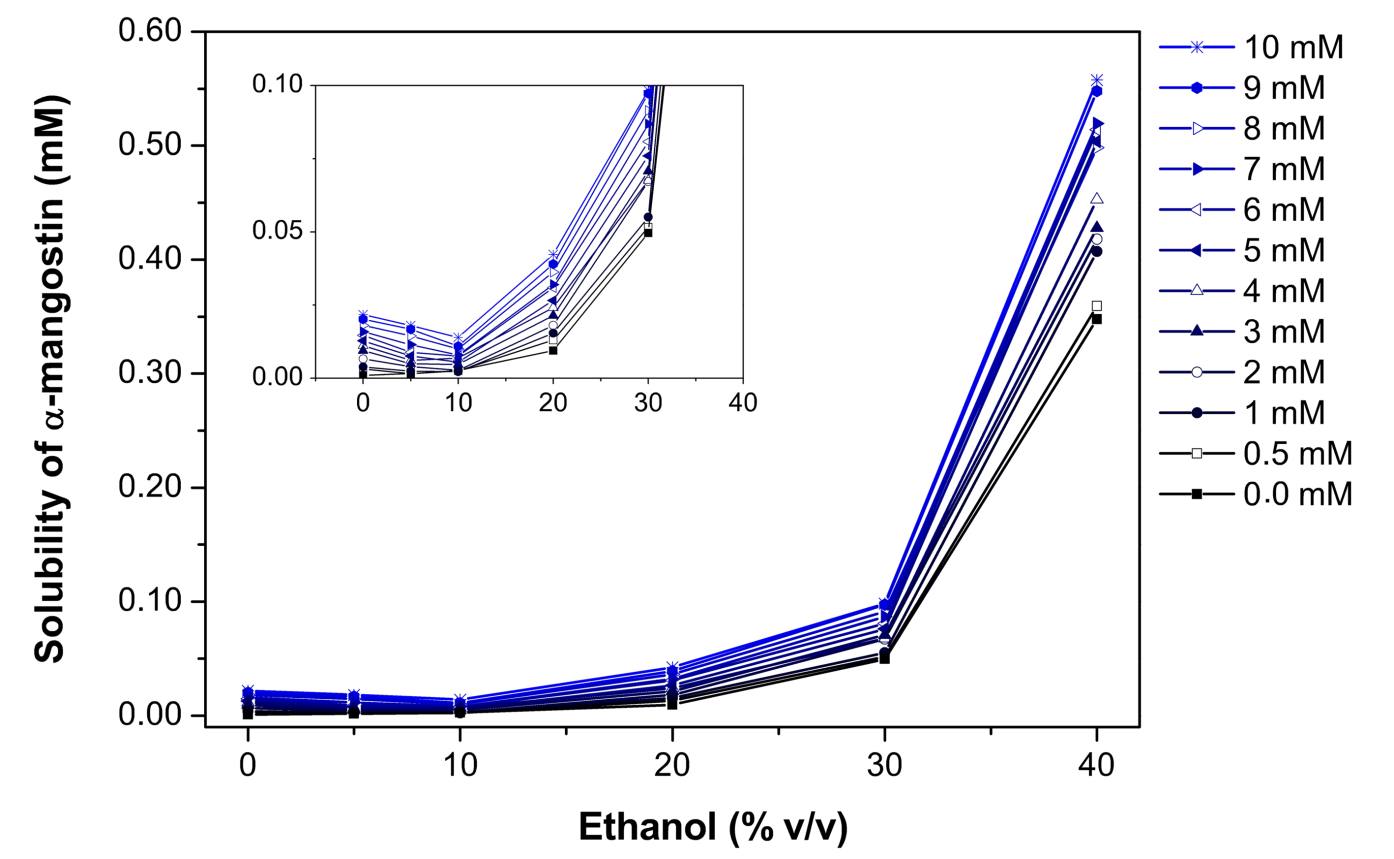
Solubility of α-mangostin as a function of ethanol concentration for different β-CD concentrations.

In this study, the phase solubility method was chosen to investigate the complexation of α-MGS and β-CD in the presence of ethanol [50]. The stoichiometry and formation constant (the equilibrium constant, *K*_b_^app^*)* can be obtained from phase solubility diagrams constructed by assessing the effect of the CD concentration on the apparent solubility of α-MGS. Figure 2 shows the α-MGS solubility increasing exponentially with ethanol concentration, as described in Equation 2. The co-solvent solubilizing power (σ, 0.36 M^−1^) was determined by plotting the logarithm of the α-MGS solubility against co-solvent concentration. The intrinsic solubility (D_u_) of α-MGS was determined to be 0.74 mM.

In the absence of ethanol, the solubility of α-MGS increases linearly with increasing β-CD concentration, up to 10 mM. The phase solubility profile can be considered to be of the A_L_ type [50] with a 1:1 β-CD and α-MGS stoichiometry as evident from the phase solubility diagram. In the absence of co-solvent, *K*_b_^app^ was equal to *K*_b_^int^ (910.91 M^−1^) and was calculated from the slope and y-intercept of the phase solubility profile.

**Figure 3:**
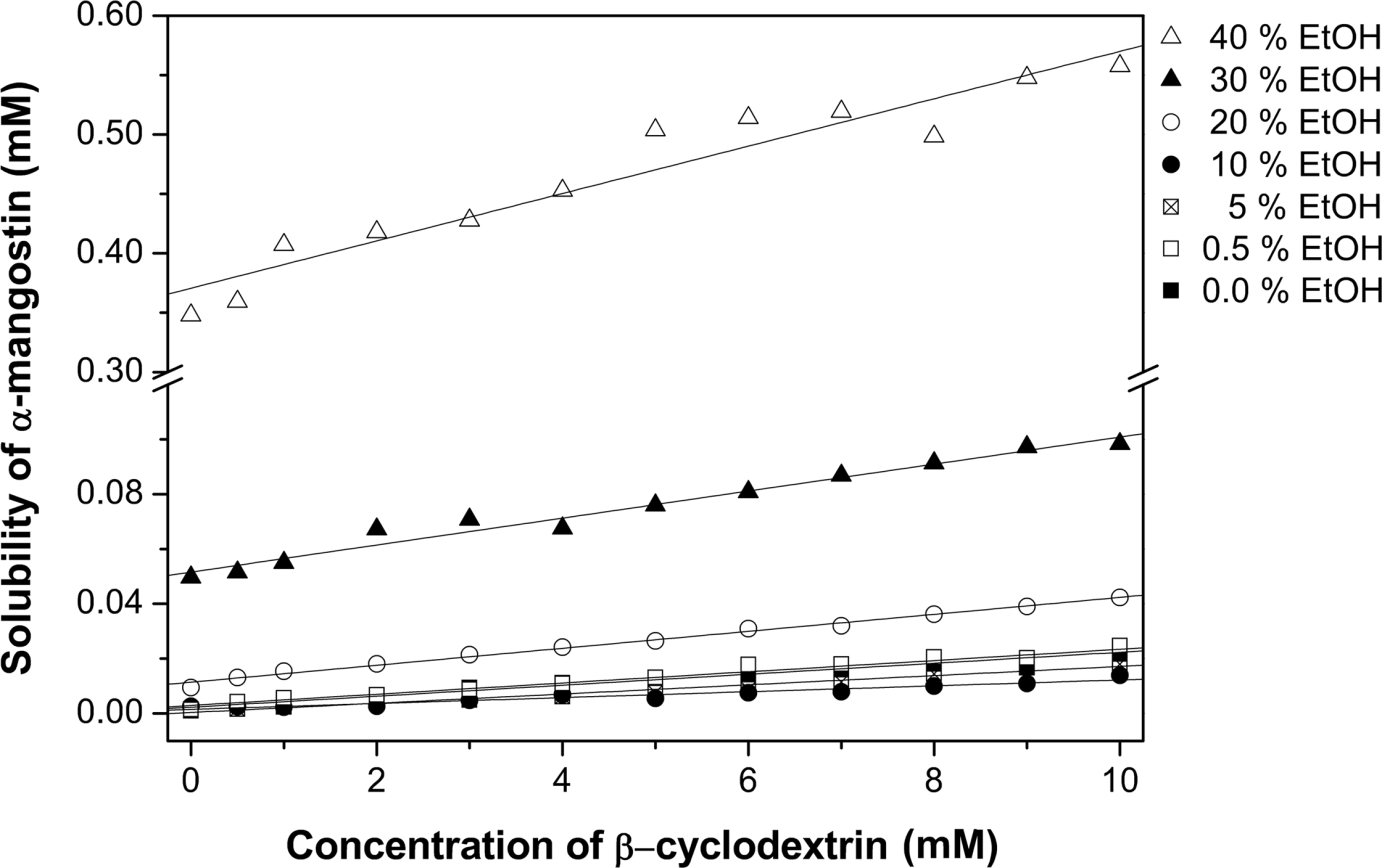
Solubility of α-mangostin as a function of β-CD for different ethanol concentrations.

According to Figure 3, the α-MGS solubility shows a linear correlation with the β-CD concentration. From considering the slope of the curve at various ethanol concentrations, it can be concluded that the solubility of α-MGS in the media containing 0.5% ethanol (% v/v) is higher than the solubility of α-MGS in pure water. This may be due to the ethanol concentration of 0.5% being not sufficient to promote ternary inclusion complex (α-MGS/β-CD/ethanol) formation. Moreover, the solubility of α-MGS decreases with increasing ethanol concentration (0.5 to 10% v/v) as a consequence of the competitive binding of ethanol to the β-CD cavity as suggested from MD simulations [22] and X-ray diffraction [51]. However, greater solubility of α-MGS was observed over a concentration range of 20–40% ethanol. The intrinsic ternary complexation constant, *K*_t_^int^, could be determined according to Equation 8, and subsequently ρ_b_ and ρ_t_ were calculated using nonlinear regression and found to be 0.27 and 0.22 M^−1^, respectively. The equilibrium constant for binary complex formation (α-MGS/β-CD) was higher than that for ternary complex formation (α-MGS/β-CD/ethanol) (Table 2). Note that no ternary complex was formed for this system. The apparent binary complexation constant, *K*_b_^app^, as a function of ethanol concentration was calculated and highlighted in Table 3. A slight decrease in complexation constant was found from 910 to 886 M^−1^ as the ethanol concentration increased. This suggests that the addition of ethanol results in increased local polarity around the α-MGS molecule, resulting in the preference for α-MGS to be located partially outside the β-CD cavity.

**Table 2:** Estimation of solubilization parameters.

Parameters	Values

D_u_ (mM)	0.74
σ (M^−1^)	0.36
ρ_b_ (M^−1^)	0.27
ρ_t_ (M^−1^)	0.22
*K*_b_^int^ (M^−1^)	910.91
*K*_t_^int^ (M^−2^)	1.61

**Table 3:** The apparent binary complexation constant, *K*_b_^app^, as a function of ethanol concentration.

Ethanol concentration (% v/v)	*K*_b_^app^ (M^−1^)

0.0	911
0.5	911
5.0	908
10.0	905
20.0	898
30.0	892
40.0	886

### Theoretical results

MD simulations were performed to investigate the effect of solvent towards the orientation and stability of the binary α-MGS/β-CD inclusion complex at the atomic level. Two conformations of inclusion complexes in water (complexes I and II in Figure S1, Supporting Information File 1) were generated, and subjected to MD simulation for 20 ns. The results implied that the displacement and mobility of the α-MGS trapped within the hydrophobic cavity of β-CD was dependent on interactions between the methoxy group presented on the narrow rim of β-CD, and the 3-methylbut-2-enyl group on the C-ring of α-MGS. Having α-MGS with its C ring located almost outside the cavity (complex II) was a preferable arrangement. On the other hand, in complex I, the secondary rim is wide enough to support two functional groups of the C-ring. Even though α-MGS has three hydroxy groups, no hydrogen bonding between guest and host molecules was detected. Thus, electrostatic interactions may not be the key factor controlling the formation of inclusion complexes; van der Waals interactions could be more important. The MM-PBSA result in Table S2, Supporting Information File 1, confirmed this assumption; the main contribution for α-MGS inclusion arises from van der Waals interactions (Δ*E*_vdW_) 7–8 fold higher than for electrostatic interactions (Δ*E*_ele_). Through summation of the solvation free energy (Δ*G*_solv_) and the entropy term (*T*Δ*S*), the predicted binding free energies (Δ*G*_bind_) of the inclusion complexes I and II are similar with values of –8.86 ± 3.25 and –9.06 ± 2.87 kcal/mol, respectively. Thus, the steric effect of the α-MGS functional groups influences only the inclusion geometry, but not the binding energy. Further details for MD simulations of the α-MGS/βCD inclusion complex in water solvation system appear in Supporting Information File 1.

#### Solvation effect on the α-MGS/β-CD inclusion complex

According to the above results, the complex II arrangement of the inclusion complex in water showed slightly higher stability. Hence, this complex was selected as the representative model, and its last snapshot was used as the initial structure for further investigations on the solvation effect by co-solvent on inclusion complex formation. Five MD simulations of the inclusion complex in aqueous solutions with different percentages of ethanol (5, 15, 30, 60 and 100% v/v) were studied for comparison with the experimental results.

#### System stability

**Figure 4:**
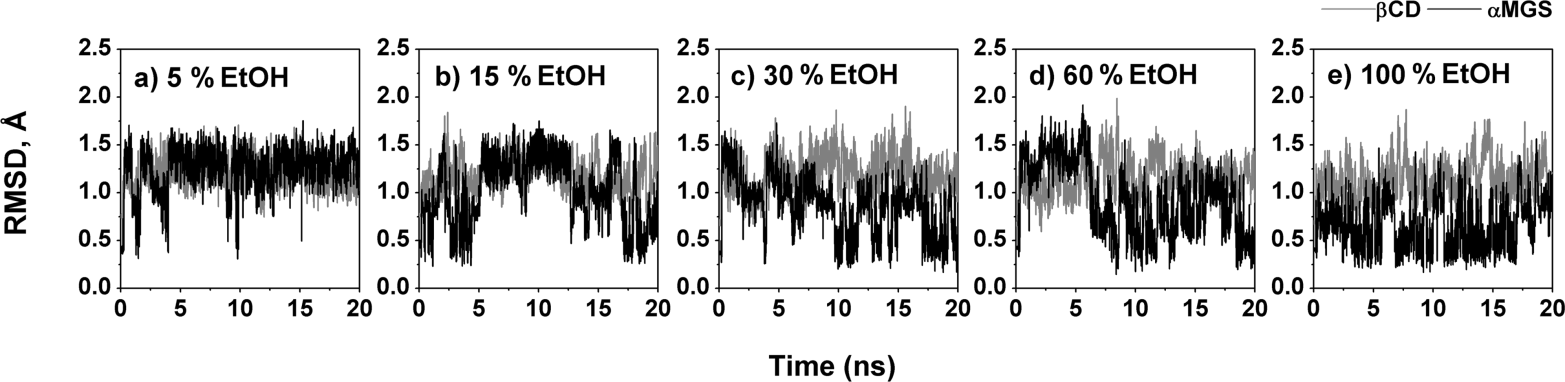
RMSD plots of β-CD (grey) and α-MGS (black) for the five systems with different ethanol percentages.

Figure 4 highlights the RMSDs of α-MGS and β-CD for five systems with increasing ethanol concentration, plotted versus simulation time. Notably, the α-MGS inside the β-CD cavity and the β-CD itself, showed more fluctuation when the ethanol percentage was raised. Adding ethanol to the aqueous solution induces greater mobility of both guest and host molecules in the inclusion complex. For a relative comparison of these situations with the inclusion complex in pure water, trajectories within the same range of the last 5 ns for the five systems focused in Figure 4 were further considered.

#### Displacement of α-mangostin

**Figure 5:**
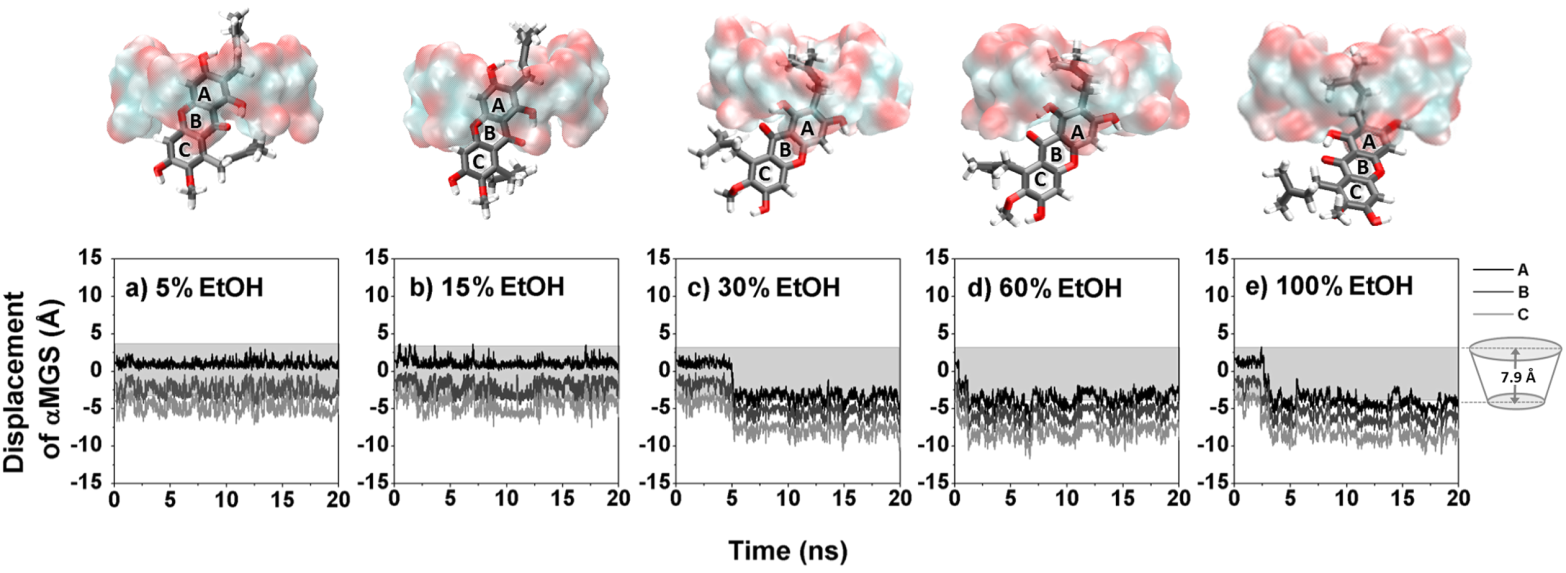
Displacement of the A–C rings of α-MGS with respect to the β-CD center of gravity for five systems having different ethanol percentages a) 5%, b) 15%, c) 30%, d) 60% and e) 100%. The last snapshot of each system is displayed above each graph.

The α-MGS displacement analysis together with the last snapshot in Figure 5 evidently shows that at low ethanol concentrations (5 and 15% v/v) α-MGS is preferentially positioned inside the hydrophobic cavity of β-CD, similar to that in water (Figure S1, Supporting Information File 1). However, the xanthone core structure of α-MGS is significantly shifted, relative to the complex formed in pure water, through the center of the β-CD cavity and thus only the A ring is partially located at the narrow rim and the 3-methylbutenyl group occupies the cavity at ≥30% v/v ethanol (Figure 5c–e). This situation consequently leads to a weak hydrogen bond (H-bond) formation between the hydroxy group (O^6^) on the A ring of α-MGS and the primary hydroxy group (O^6^) on the narrow edge of the β-CD (Table 4). The H-bond strength showed an enhancement as a function of alcohol concentration (% H-bond of 32, 60 and 77 for 30, 60 and 100% v/v ethanol, respectively), which likely promoted electrostatic interactions between the α-MGS and β-CD molecules (∆*E*_ele_ in Table 5). The data obtained also suggested that, at high alcohol content ≥30% v/v, ethanol greatly stabilized the hydrophobicity of aromatic ring outside the β-CD cavity as it can be seen by the co-solvent accessibility towards the trapped α-MGS (discussed below).

**Table 4:** Percentage of hydrogen bond (% H-bond) formed between the hydroxy groups of α-MGS and the β-CD molecules, O^6^–H^6^(α-MGS)···O^6^(β-CD), in six inclusion complexes.

System	% H-bond

water	–
5% v/v EtOH	–
15% v/v EtOH	–
30% v/v EtOH	32
60% v/v EtOH	60
EtOH	77

#### Radial distribution function analysis

**Figure 6:**
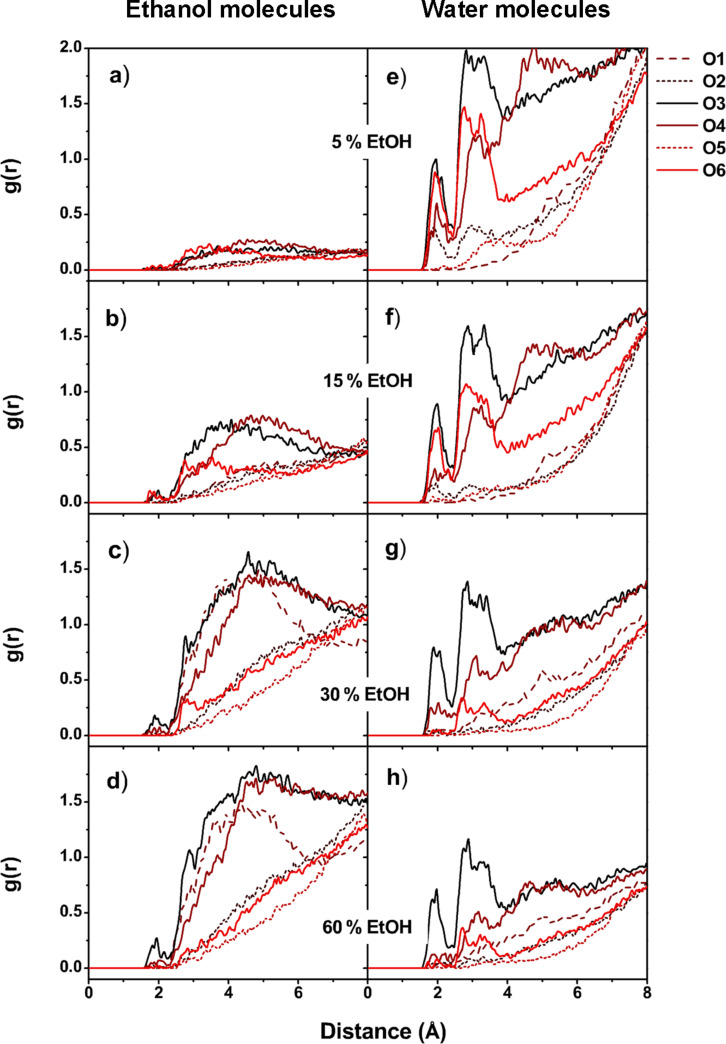
Radial distribution functions (RDF) of (a–d) ethanol, and (e–h) water molecules around the oxygen atoms of α-MGS on complexation with β-CD at different ethanol percentages.

To probe the influence of ethanol co-solvent towards α-MGS occupation within the β-CD cavity, the radial distribution function (RDF or *g_ij_*(*r*)) was used to monitor the solvation of water and/or ethanol around the α-MGS in the formed inclusion complexes. The *g_ij_*(*r*) was calculated as a function of the ethanol or water oxygen atom *j* within a spherical radius of *r* from the α-MGS heteroatom (oxygen atom *i*). The RDF results of ethanol and water co-solvation are shown in the left and right columns of Figure 6, respectively. The integration number, *n*(*r*), of solvent molecules are presented in Table S3 of Supporting Information File 1. RDFs of systems with pure water, and pure ethanol solvation, are presented in Figure S2 of Supporting Information File 1.

For the systems in water, and with low ethanol concentrations (5 and 15% v/v EtOH), the xanthone ring of the α-MGS is mostly localized within the β-CD cavity, with its functional groups located close to the β-CD rims, as already discussed. Thus, the sharp peaks of water molecules noticeably appear around 2.2 Å and 3.1 Å of the O^2^, O^3^, O^4^ and O^6^ atoms of the α-MGS (see Figure 6), which represent the first and second solvation shells with the numbers of solvated water molecules ranked in order of O^3^ > O^6^ > O^4^ > O^2^. In contrast, no sharp peak for ethanol solvation appeared within ≈3 Å of all six oxygen atoms of α-MGS, suggesting that only a very small amount of ethanol was able to access the mostly entrapped α-MGS at low alcohol concentrations.

**Figure 7:**
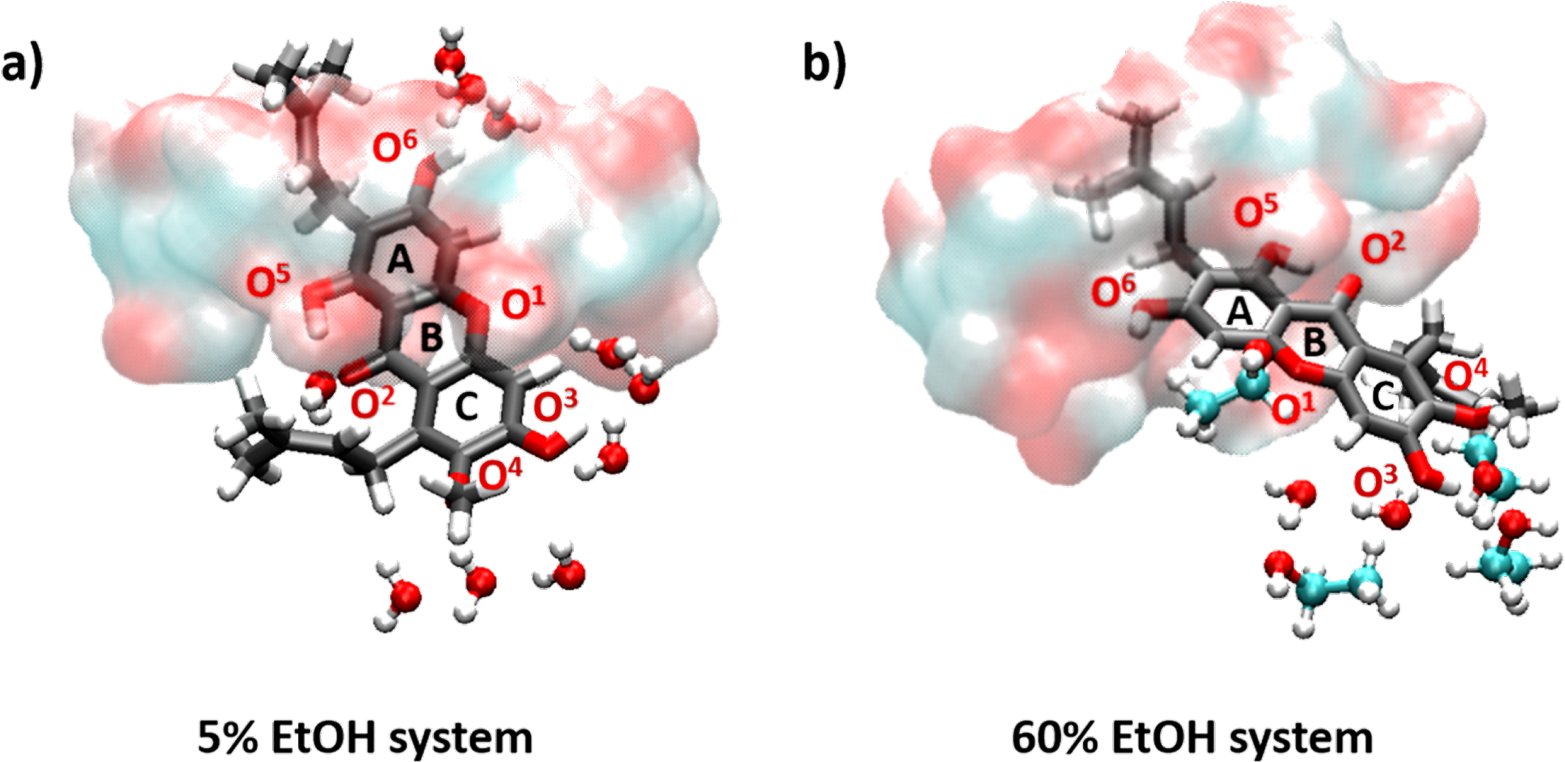
Snapshots of solvation around heteroatoms of α-MGS/β-CD for systems containing 5% and 60% v/v ethanol.

At higher alcohol content (≥30% v/v ethanol), the number of water molecules in the first solvation shell around the heteroatoms of α-MGS dramatically decreased, especially for the O^6^ and O^2^ atoms. This is a result of partial displacement of α-MGS from the β-CD cavity. When the percentage of ethanol solvation increases, only the 3-methylbut-2-enyl group and a portion of the A-ring are located inside the β-CD cavity, whilst the B- and C-rings are almost completely displaced. For this reason, O^6^ is shielded by the narrow rim of β-CD while O^1^, O^3^ and O^4^ are exposed to solvent molecules (Figure 7). The first solvation shell of ethanol at 60% v/v concentration appears around 2 Å from O^3^ of α-MGS, but the *n*(*r*) of ethanol molecules (0.2) is lower than the *n*(*r*) of water molecules (0.4) for the same shell as shown in Table S3 of Supporting Information File 1. The number of solvated ethanol molecules increases in the secondary solvation shell (≈3 Å) from O^3^, O^1^ and O^4^ atoms with *n*(*r*) values of 2.2, 1.5 and 1.0, respectively. Compared to the *n*(*r*) of water molecules in the secondary solvation shell of O^3^ (2.5), it is conceivable that, in instances of co-solvation, a lower degree of water solvation is well compensated by the higher accessibility of ethanol molecules to the α-MGS heteroatoms in the secondary solvation shell. It is worth noting that the O^3^ atom is the most attractive site for solvation molecules because water and ethanol molecules always solvate around 2 Å from O^3^, compared to other oxygen atoms in α-MGS.

#### Binding energy analysis

Based on the MM-PBSA approach, the binding free energies of the α-MGS/β-CD complexes at various EtOH concentrations were predicted. The decomposition of free energy into additive contributions has potential to provide relationship between structure and binding affinity as well as the solvation effects. Theoretical basis of solvation free energy decomposition and the Free Energy Perturbation (FEP) formalism allowing additive for free-energy contributions arising from different types of interaction were well defined by Bren et al. [52–53]. To evaluate the solvation effect in this work, the binding free energies were decomposed in Table 5.

**Table 5:** MM-PBSA binding free energies (kcal/mol) and their energy components for α-MGS/β-CD complexes at different EtOH concentrations.

		EtOH concentration (% v/v)					
	
		0%	5%	15%	30%	60%	100%

Δ*E*_ele_		−4.61 ± 2.67	−4.30 ± 2.35	−4.99 ± 3.07	−8.69 ± 4.50	−10.17 ± 4.35	−10.20 ± 3.84
Δ*E*_vdW_		−37.04 ± 1.93	−37.46 ± 2.55	−36.14 ± 2.53	−28.36 ± 4.43	−26.98 ± 4.10	−22.39 ± 3.72
Δ*E_MM_*	(1)	−41.65 ± 3.22	−41.76 ± 3.59	−41.13 ± 4.02	−37.05 ± 5.77	−37.15 ± 5.80	−32.58 ± 5.41
Δ*G_nsolv_*		−4.53 ± 0.17	−4.49 ± 0.21	−4.48 ± 0.20	−4.01 ± 0.31	−3.91 ± 0.30	−3.54 ± 0.32
Δ*G_psolv_*		23.83 ± 3.80	23.03 ± 3.75	22.21 ± 4.10	18.51 ± 3.57	18.86 ± 3.53	16.70 ± 2.95
Δ*G_solv_*	(2)	19.30 ± 3.72	18.54 ± 3.64	17.73 ± 3.99	14.50 ± 3.42	14.94 ± 3.39	13.16 ± 2.75
Δ*G*_psolv_ + *E_ele_*		19.22 ± 3.00	18.73 ± 3.13	17.22 ± 3.17	9.83 ± 3.3	8.68 ± 3.19	6.50 ± 3.18
Δ*G*_nsolv_ + *E_vdW_*		−41.57 ± 2.10	−41.95 ± 2.76	−40.62 ± 2.73	−32.37 ± 4.74	−30.89 ± 4.40	−25.93 ± 4.04
−TΔ*S*	(3)	13.29 ± 2.72	13.00 ± 2.73	12.88 ± 2.92	13.04 ± 2.44	13.31 ± 3.10	12.46 ± 2.51

Δ*G_bind _*_ (1)+(2)+(3)_		−9.06 ± 2.87	−10.21 ± 2.84	−10.51 ± 2.93	−9.51 ± 3.19	−8.90 ± 3.56	−6.96 ± 3.14

In line with the hydrogen bond analysis, the binding energy in terms of electronic interactions (Δ*E_ele_*) significantly increased from > −5 kcal/mol in pure water and low ethanol concentrations to −8.69, −10.17 and −10.20 kcal/mol in 30, 60 and 100% v/v ethanol. By contrast, the van der Waals energy contribution (Δ*E*_vdW_) was reduced by ≈10–15 kcal/mol due to almost total displacement of the α-MGS xanthone ring from the β-CD cavity via the primary rim. However, the magnitude of Δ*E*_ele_ was lower than Δ*E*_vdW_, which is known to be the main factor governing the stability of CD inclusion complexes [35]. By considering the solvation effect, we found that the presence of ethanol molecules can enhance the solvation energy (Δ*G_solv_)* of the inclusion complex, as seen by a reduction in Δ*G*_solv_ at high ethanol percentages. In contrast, the entropies of all systems were likely similar (*−T∆S* of ≈13 kcal/mol). After combining the interaction energy (1), solvation (2) and entropy (3) terms, the binding affinity of the α-MGS/β-CD complexation at 0–60% v/v ethanol almost steady at the range of −9.06 to −8.90 kcal/mol. This is because increases in Δ*E*_MM_ are compromised by a lowering of Δ*G*_solv_. Moreover, the inclusion complex in pure ethanol is less stable than that in pure water, by ca. 2 kcal/mol. By taken altogether, the addition of ethanol mainly affects the displacement and solvation accessibility of α-MGS in the inclusion complex, rather than its binding affinity in term of the total binding free energy. These results are in line with our experimental study where increasing the ethanol percentage does not dramatically reduce the *K*_b_^app^ of the binary inclusion complex.

## Conclusion

In this study the effect of water/ethanol co-solvation systems on the formation of α-MGS/β-CD complexes has been investigated. From experimental work, a mathematical model was used to explain complex formation in relation to phase solubility. From the equilibrium constant calculation it was found that the inclusion complex is still a binary complex, even in the presence of ethanol. When the ethanol concentration was higher than 10% v/v, the solubility of α-MGS was enhanced. Besides, increasing the ethanol concentration resulted in slight decreases in the α-MGS/β-CD complexation constant. The MD simulation results indicated that the dynamics property of α-MGS in respect to the β-CD cavity axis, the solvent accessibility towards the encapsulated α-MGS and the binding affinity of the inclusion complex depend on the ethanol concentrations. At high ethanol concentrations (>30% v/v), the stability of the hydrophobic aromatic ring of the α-MGS outside the inclusion cavity was promoted resulting in a reduced binding interaction but enhanced solubility of the α-MGS/β-CD inclusion complex. As a compromise between those two factors, interaction energy and solvation free energy, the total binding free energy of the α-MGS/β-CD was slightly reduced when the ethanol percentage was increased. In conclusion, the presence of ethanol enhances the solubility of α-MGS and its inclusion complex, α-MGS/β-CD, with effects on the binding affinity with β-CD being dependent on the co-solvent concentration.

## Supporting Information

File 1 Additional data.
