# Supplementary material for: Co-solvation effect on the binding mode of the α-mangostin/β-cyclodextrin inclusion complex
Source: Beilstein J Org Chem. 2015 Nov 25;11:2306–17. doi: 10.3762/bjoc.11.251 (PMC4685766; doi:10.3762/bjoc.11.251)
Supplement: File 1 — Additional data. [file Beilstein_J_Org_Chem-11-2306-s001.pdf]

Supporting Information

for

Co-solvation effect on the binding mode of the α-mangostin/β-cyclodextrin inclusion complex

Chompoonut Rungnim[1], Sarunya Phunpee[1], Manaschai Kunaseth[1], Supawadee Namuangruk[1], Kanin Rungsardthong[2], Thanyada Rungrotmongkol[*,‡,3,4,§] and Uracha Ruktanonchai[*,‡,1,]

Address: [1]National Nanotechnology Center (NANOTEC), National Science and Technology Development Agency (NSTDA), Pathumthani 12120, Thailand, [2]Faculty of Pharmacy Thammasat University, Rangsit Center, Pathumthani 12120 Thailand, [3]Structural and Computational Biology Unit, Department of Biochemistry, Faculty of Science, Chulalongkorn University, Bangkok 10330, Thailand and [4]Ph.D. Program in Bioinformatics and Computational Biology, Faculty of Science, Chulalongkorn University, Bangkok 10330, Thailand

Email: Uracha Ruktanonchai - uracha@nanotec.or.th

Email: Uracha Ruktanonchai - t.rungrotmongkol@gmail.com,

*Corresponding Author

[‡]These authors contributed equally to this work

[§]Fax:+66(0)2564-6985; Tel.+66(0)2564-6552,

[¶]Fax:+66(0)2218-5418; Tel.+66(0)2218-5426

Additional data

1. Parameters of α-mangostin

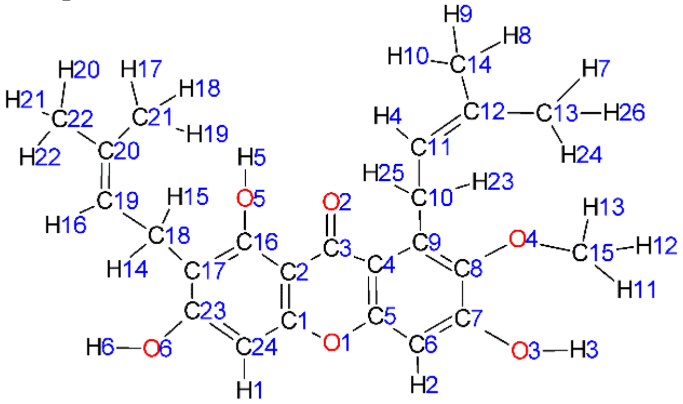

Table S1: Atom types and partial charges of α-mangostin ($C_{24}H_{26}O_6$)

Name	Type	q	Name	Type	q	Name	Type	q
C13	CT	−0.2454	O5	OH	−0.5789	H5	CA	−0.7066
H24	HC	0.0774	H5	HO	0.4428	H1	HA	0.2645
H26	HC	0.0774	C17	CA	−0.3465	O6	OH	−0.6283
H7	HC	0.0774	C18	CT	0.1081	H6	HO	0.4754

Name	Type	q	Name	Type	q	Name	Type	q
C12	CM	0.1632	C19	CM	−0.429	C1	CA	0.5328
C14	CT	−0.2454	C20	CM	0.1938	O1	OS	−0.3212
H8	HC	0.0774	C21	CT	−0.3281	C5	CA	0.4579
H9	HC	0.0774	H17	HC	0.0989	C6	CA	−0.612
H10	HC	0.0774	H18	HC	0.0989	H2	HA	0.2511
C11	CM	−0.4282	H19	HC	0.0989	C7	CA	0.4545
H4	HA	0.1947	C22	CT	−0.3281	O3	OH	−0.6202
C10	CT	0.0641	H20	HC	0.0989	H3	HO	0.4765
H23	HC	0.0763	H21	HC	0.0989	C8	CA	0.0344
H25	HC	0.0763	H22	HC	0.0989	O4	OS	−0.3791
C9	CA	0.0394	H16	HA	0.2054	C15	CT	0.2301
C4	CA	−0.4221	H14	HC	0.068	H11	HC	0.0188
C3	C	0.7764	H15	HC	0.068	H12	HC	0.0188
O2	O	−0.5921	C23	CA	0.548	H13	HC	0.0188
C2	CA	−0.5643	C16	CA	0.4603			

2. Inclusion complex of α-MGS/β-CD in a water solvation system

To explore the preferential orientation of α-MGS inside the β-CD cavity, two conformations of inclusion complexes in water (complexes I and II in **Figure S1**) were generated and subjected to MD simulation for 20 ns. For inclusion complex I, the A ring of α-MGS was placed close to the narrow rim of β-CD (O^6 region). On the other hand, the α-MGS in complex II was oriented in the opposite direction.

2.1 System stability

The RMSDs of the α-MGS and β-CD, relative to their initial coordinates, were monitored along the 20 ns of MD simulation and are shown in **Figure S1 (a**,**b)**. During the first

15 ns, the α-MGS in complex I was positioned closer to the starting structure (0.79 ± 0.31 Å) than that of complex II (1.2 3± 0.28 Å). Meanwhile, the RMSD of β-CD in both complexes was steady at ≈1.3 Å. Since two systems apparently reached their equilibration states after 15 ns, the trajectories from the last 5 ns were extracted for structural and binding energy analysis.

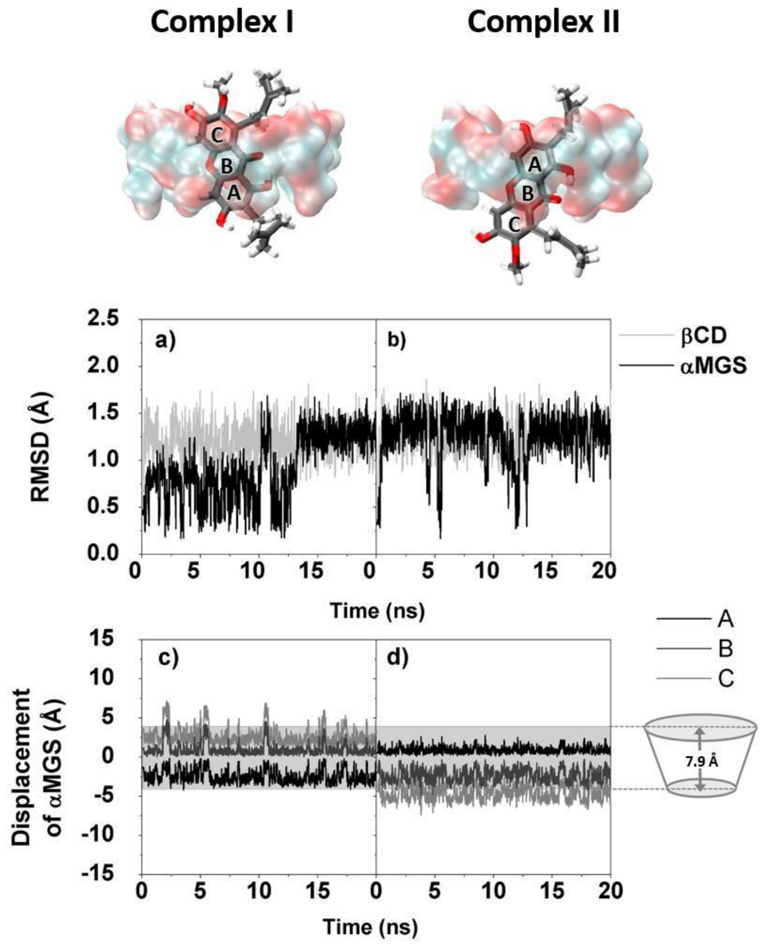

Figure S1: (a,b) RMSD plots of α-MGS and β-CD with respect to their initial structures, and (c,d) displacement plots of the α-MGS rings A–C versus simulation time for the inclusion complexes I and II. The last snapshot structures are depicted above the graphs, where the light blue shade represents the β-CD cavity.

2.2 Displacement of α-mangostin

From the displacement analysis in **Figure S1 (c-d)**, the α-MGS in complex I fluctuated inside the β-CD cavity while its inclusion in complex II is more stable with only slight oscillation. The α-MGS in complex II is also located deeper in the cavity and more towards the narrow rim of β-CD, as seen by the displacements of the A- and C-rings (approximately at 0.9 and −4.7 Å, respectively).

2.3 Binding energy analysis

MM-PBSA binding free energies of the α-MGS/β-CD complexes I and II are presented in **Table S1**. It can be seen that the binding affinities of the two forms are likely comparable (ΔG_{bind} of ≈9 kcal/mol).

Table S2: MM-PBSA binding free energy and the energy components (kcal/mol) for the α-MGS/β-CD complexes I and II

	**Complex I**	**Complex II**
ΔE_{ele}	−5.33 ± 3.34	−4.61 ± 2.67
ΔE_{vdW}	−38.96 ± 2.86	−37.04 ± 1.93
ΔE_{MM} (1)	-44.29 ± 4.16	-41.65 ± 3.22
ΔG_{nsolv}	−4.68 ± 0.19	−4.53 ± 0.17
ΔG_{psolv}	27.55 ± 4.26	23.83 ± 3.80
ΔG_{solv} (2)	22.87 ± 4.22	19.30 ± 3.72
$\Delta G_{psolv} + E_{ele}$	18.94 ± 2.14	19.22 ± 3.00
$\Delta G_{nsolv} + E_{vdW}$	−43.64 ± 1.53	−41.57 ± 2.10
$-T\Delta S$ (3)	12.56 ± 2.72	13.29 ± 2.72
ΔG_{bind} (1) + (2) + (3)	−8.86 ± 3.25	−9.06 ± 2.87

3. Solvation accessibility

3.1 Solvation accessibility in pure water or ethanol

The RDF plots of the oxygen atoms of α-MGS complexed with β-CD in pure water, and ethanol, are presented in **Figure S2**. The first solvation shell in water solvation system appears around 2 Å from oxygen atoms (O^2, O^3, O^4 and O^6) of the encapsulated α-MGS. Differentially, a much lower accessibility of ethanol is observed in the same solvation shell around the O^3 and O^2 atoms.

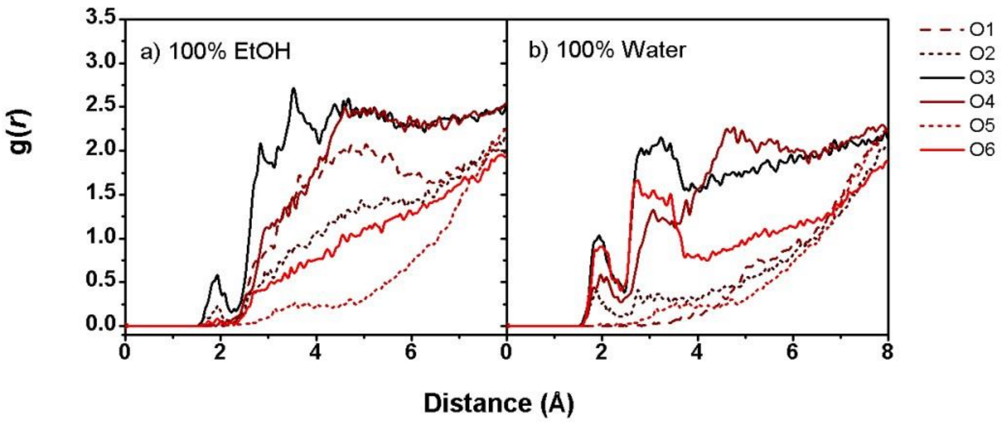

Figure S2: Radial distribution function (RDF, *g(r)*) of (a) ethanol, and (b) water oxygens towards the ligand oxygens (O^1–O^6) of α-MGS/β-CD complex.

3.2 Solvation accessibility in co-solvent

Table S3: Integration number, *n(r)*, of the first (2.2 Å) and second solvation shells (3.1 Å) around the heteroatoms of α-MGS in each system

System	*n(r)* of water molecules		*n(r)* of ethanol molecules	
	1st solvation shell	2nd solvation shell	1st solvation shell	2nd solvation shell
**Water**				
O^1	0.0	0.0	-	-
O^2	0.2	0.9	-	-
O^3	0.7	4.9	-	-
O^4	0.4	2.6	-	-
O^5	0.0	0.2	-	-
O^6	0.7	4.1	-	-
**5 %v/v Ethanol**				
O^1	0.0	0.0	0.0	0.0
O^2	0.2	1.0	0.0	0.0
O^3	0.7	4.6	0.0	0.2
O^4	0.4	2.4	0.0	0.2
O^5	0.0	0.2	0.0	0.0
O^6	0.6	3.5	0.0	0.4
**15 %v/v Ethanol**				
O^1	0.0	0.0	0.0	0.1
O^2	0.1	0.4	0.0	0.2
O^3	0.6	3.7	0.0	1.0
O^4	0.2	1.7	0.0	0.6
O^5	0.0	0.2	0.0	0.1
O^6	0.4	2.7	0.0	0.7
**30 %v/v Ethanol**				

System	*n(r)* of water molecules		*n(r)* of ethanol molecules	
	**1st solvation shell**	**2nd solvation shell**	**1st solvation shell**	**2nd solvation shell**
O^1	0.0	0.3	0.0	1.5
O^2	0.0	0.1	0.0	0.2
O^3	0.6	3.2	0.1	1.8
O^4	0.2	1.4	0.0	1.0
O^5	0.0	0.1	0.0	0.1
O^6	0.0	0.6	0.0	0.6
**60 %v/v Ethanol**				
O^1	0.0	0.3	0.0	1.5
O^2	0.0	0.2	0.0	0.3
O^3	0.4	2.5	0.2	2.2
O^4	0.1	0.8	0.0	1.0
O^5	0.0	0.0	0.0	0.1
O^6	0.0	0.6	0.0	0.3
**Ethanol**				
O^1	-	-	0.0	1.7
O^2	-	-	0.1	1.2
O^3	-	-	0.3	4.1
O^4	-	-	0.0	2.0
O^5	-	-	0.0	0.7
O^6	-	-	0.0	0.9
